# Rif1 acts through Protein Phosphatase 1 but independent of replication timing to suppress telomere extension in budding yeast

**DOI:** 10.1093/nar/gky132

**Published:** 2018-02-26

**Authors:** Sylwia Kedziora, Vamsi K Gali, Rosemary HC Wilson, Kate RM Clark, Conrad A Nieduszynski, Shin-ichiro Hiraga, Anne D Donaldson

**Affiliations:** 1 Institute of Medical Sciences, University of Aberdeen, Foresterhill, Aberdeen AB25 2ZD, Scotland, UK; 2 Sir William Dunn School of Pathology, University of Oxford, South Parks Road, Oxford OX1 3RE, UK

## Abstract

The Rif1 protein negatively regulates telomeric TG repeat length in the budding yeast *Saccharomyces cerevisiae*, but how it prevents telomere over-extension is unknown. Rif1 was recently shown to control DNA replication by acting as a Protein Phosphatase 1 (PP1)-targeting subunit. Therefore, we investigated whether Rif1 controls telomere length by targeting PP1 activity. We find that a Rif1 mutant defective for PP1 interaction causes a long-telomere phenotype, similar to that of *rif1Δ* cells. Tethering PP1 at a specific telomere partially substitutes for Rif1 in limiting TG repeat length, confirming the importance of PP1 in telomere length control. Ablating Rif1–PP1 interaction is known to cause precocious activation of telomere-proximal replication origins and aberrantly early telomere replication. However, we find that Rif1 still limits telomere length even if late replication is forced through deletion of nearby replication origins, indicating that Rif1 can control telomere length independent of replication timing. Moreover we find that, even at a *de novo* telomere created after DNA synthesis during a mitotic block, Rif1–PP1 interaction is required to suppress telomere lengthening and prevent inappropriate recruitment of Tel1 kinase. Overall, our results show that Rif1 controls telomere length by recruiting PP1 to directly suppress telomerase-mediated TG repeat lengthening.

## INTRODUCTION

Telomeres play a crucial role in ensuring genome stability, by protecting the chromosome ends and preventing their gradual erosion in successive cell cycles due to the end-replication problem (reviewed by (1)). Telomeres maintain their length by periodically promoting recruitment of telomerase, the specialized reverse transcriptase that extends the TG-rich terminal repeat sequences. Many central telomere regulators were initially identified in the budding yeast *Saccharomyces cerevisiae*, which provides a powerful model system for understanding telomere length control. In *S. cerevisiae* cells, telomerase is preferentially recruited to the shortest TG tracts that are most in need of extension, as outlined in recent reviews describing the mechanisms controlling telomere lengthening (2,3). Briefly, short telomeres are recognized by the Mre11–Rad50–Xrs2 (MRX) complex (4), which recruits Tel1 kinase through interaction with Xrs2: binding of Tel1 and the MRX complex appear to be mutually reinforcing (5). Several studies have shown that Tel1 is central for the specific recruitment of telomerase to short telomeres (6–11). Kinase activity of Tel1 is important for telomere extension (12), and Cdc13 was identified as a likely phosphorylation target. However, a Cdc13 mutant allele with all Tel1 consensus phosphosites mutated did not lead to the expected short telomeres (13), so that the Tel1 target(s) and phosphorylation sites important for TG extension in *S. cerevisiae* have not been conclusively identified (2). Consistent however with a central role for Tel1 and the MRX complex in promoting telomere lengthening, *tel1Δ, mre11Δ, rad50Δ* and *xrs2Δ* mutants all have very short telomeres.

The Rif1 and Rif2 (Rap1-interacting factors) were originally identified in *S. cerevisiae* as negative regulators of telomere length that are recruited to telomeres by the C-terminal domain of Rap1, which directly recognizes the TG repeats (14,15). Deleting either *RIF1* or *RIF2* leads to substantial TG repeat elongation, mediated by inappropriate telomerase recruitment to TG tracts not in need of extension. These discoveries provided the foundation for a ‘protein-counting’ model of telomere length control (16), in which the recruitment of a sufficient number of Rap1 and Rif1/2 molecules suppresses telomerase recruitment at normal-length telomeres not in need of elongation. While the exact mechanisms through which they prevent telomerase recruitment remain unclear, Rif1 and Rif2 appear to act through different pathways as their effects on telomere length are additive (i.e. telomeres are somewhat long in a *rif2Δ* mutant, very long in a *rif1Δ* mutant and longer still in a *rif1Δ rif2Δ* double mutant). Rif1 and Rif2 may affect telomere length partly by competing with Sir proteins for binding to the Rap1 C-terminus, since Sir proteins promote telomerase recruitment (17,18). However, the *rif1Δ* mutation still causes telomere lengthening when the Sir-mediated pathway of telomerase recruitment is ablated (17), implying this is not the only, or even the principal, pathway through which Rif1 suppresses lengthening by telomerase. Indeed, tethered Rif1 represses inappropriate Tel1 recruitment to adjacent telomeric sequence (5), but without affecting recruitment of MRX components.

**Figure 1.**
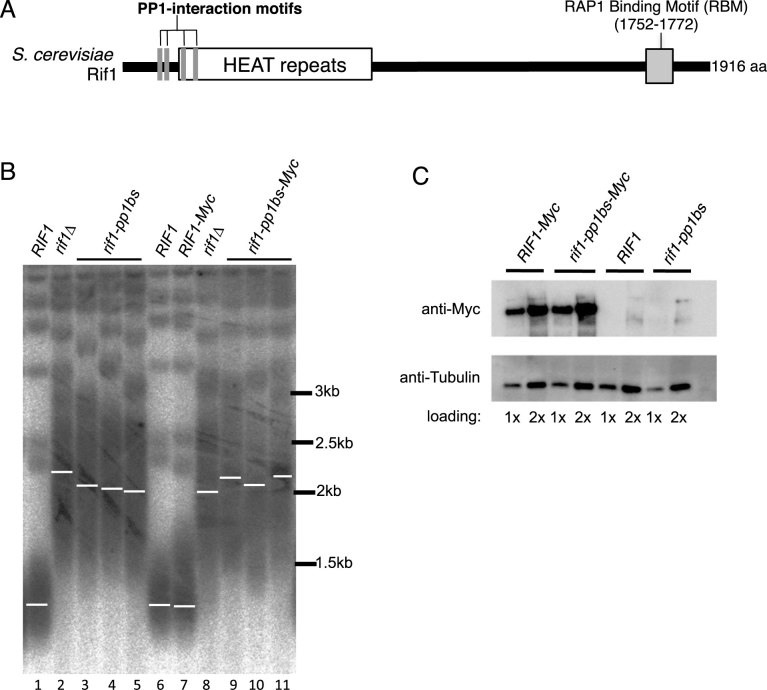
Telomere length is elongated in *rif1–pp1bs* mutants similar to *rif1Δ*. (**A**) Schematic illustration of Rif1 protein domains. The four PP1 interaction motifs mutated in the *rif1–pp1bs* mutant are indicated. (**B**) Th*e rif1–pp1b*s mutation causes long telomeres (lanes 3–5 and 9–11) indicative of a role for PP1 in telomere length control. Genomic DNA was digested with XhoI, and Y’ terminal fragments were detected using a telomeric TG probe. Three isolates each o*f rif1–pp1b*s an*d rif1–pp1bs-My*c are shown. All strains in BY4741 background. (**C**) Western blot analysis confirms similar protein levels of Rif1-Myc and Rif1–pp1bs-Myc proteins. Strains used (B and C): SHY201, SMKY28, SMKY29, ASY26, SMKY30 and SMKY31.

Recent studies have however shed significant light on molecular mechanisms through which the Rif1 protein operates, at telomeres and in other functional contexts (19). It has emerged that the role of yeast Rif1 is not limited to telomere control, and that it also has important effects on other cellular functions including DNA replication. Specifically, Rif1 was found to prevent premature activation of replication origins in normally late-replicating chromosomal domains, including telomere-proximal regions (20–23). Rif1 controls origin activation by directing the activity of Protein Phosphatase 1 (PP1, encoded in *S. cerevisiae* by the gene *GLC7* (24)). Like most phosphatases, PP1 has intrinsically low specificity and must be targeted to biologically relevant targets by a PP1 substrate-targeting subunit. Rif1 acts as such a PP1 substrate-targeting subunit to direct the dephosphorylation of subunits of the Minichromosome Maintenance (MCM) complex, preventing premature activation of the MCM complex replicative helicase function. To fulfill this role, Rif1 interacts with PP1 through a series of N-terminal PP1 interaction motifs (Figure 1A), conforming to the so-called ‘SILK’ and ‘RVxF’ consensus sequences well-established as mediating PP1 interaction (25). This function of Rif1 in controlling DNA replication is evolutionarily conserved (26–28).

The discovery that Rif1 is a conserved PP1 substrate-targeting subunit prompted us to examine whether Rif1 also acts through PP1 to control telomere length. Here we show that interaction of Rif1 with PP1 is indeed essential for suppression of TG repeat elongation, and that tethering PP1 can partly bypass the need for Rif1 to prevent telomere over-elongation. It has been proposed that the effect of Rif1 on telomere length might be mediated through its effect on nearby replication origins (29), because at short telomeres with limited Rif1, earlier replication could potentially facilitate the recruitment or retention of telomerase (19,30). Here we show that, contrary to such models, Rif1 can affect telomere length without regulating telomere replication time. We find instead that Rif1–PP1 directly suppresses Tel1 recruitment and inappropriate telomere lengthening, and can exert this role immediately even at a de novo telomere induced during a mitotic block.

## MATERIALS AND METHODS

### Yeast strains and plasmids

The yeast strains used are listed in Supplementary Table S1. Yeast gene deletions and epitope tagging were made by the one-step polymerase chain reaction (PCR) gene replacement method (31,32) and confirmed by colony PCR across the junction sites. To delete *ARS608.5*, a 26 bp genomic segment containing the ARS Consensus Sequence for *ARS608.5* (A. Wolstenholme and C. Nieduszynski (personal communication)) was replaced by a DNA fragment containing *ADE2* gene, by means of one-step PCR gene replacement.

Plasmids used in this study are listed in Supplementary Table S2. The pSMK2 plasmid expressing LexA-Glc7 in-frame fusion protein was created by PCR-amplifying the *GLC7* cDNA sequence (24) using primers SMKY53 and SMKY54, and cloned into SmaI-digested pAT4 plasmid (33,34) using In-Fusion HD cloning system (Clontech).

The pSMK5 plasmid expressing LexA-Glc7-H124A mutant protein was created by inverse PCR mutagenesis (35) using primers SMK154 and SMK155 with plasmid pSMK2 as a template, followed by *Dpn*I digestion and *in vivo* circularization in *Escherichia coli*. Correct plasmid clones were selected by diagnostic PCR and restriction digestion. Introduction of the designed mutation and the absence of other mutations or rearrangements was confirmed by sequencing.

Custom synthesis and cloning of cDNA of human PPP1R2 isoform 2 (Genbank accession number NM_006241.7) into pESC-URA vector (under the control of *GAL10* promoter) was carried out by GenScript to create the pESC-URA-PPP1R2 plasmid. DNA Sequence of the plasmid is available upon request.

### Analysis of telomere length

Telomere length was analyzed essentially as described (36). Restriction enzymes and probes used are described in figure legends. The probe used for Figure 3A and B was prepared by PCR amplification from genomic DNA using primers SMK71 and SMK72 (see Supplementary Table S3 for primers used). Probe DNA fragment used in Figure 6C and Supplementary Figure S6B was prepared by PCR amplification using primers SMK123 and SMK125.

### Chromatin immunoprecipitation (ChIP)

Chromatin immunoprecipitation (ChIP) was performed essentially as described (37) with modifications: cells were disrupted using a FastPrep-24 bead beater (MP Biomedicals), and sonication of DNA was performed using a Bioruptor (Diagnode). Antibodies used were mouse monoclonal anti-Myc antibody [9E11] (Abcam, ab56) and rabbit polyclonal anti-HA antibody (Abcam, ab9110). Dynabeads Protein-G (Dynal) were used.

### Quantitative real-time PCR

Immunoprecipitates were analyzed by quantitative real-time PCR using LightCycler 480 (Roche) with LightCycler 480 SYBR Green master mix (Roche). ChIP efficiencies were calculated based on the differences between Ct values of ChIP samples and input samples, taking account of amplification efficiencies of each primer pair. PCR primers used are: M2245 and M2246 for telomere *VI-R*; M1367 and M1368 for *PAC2* locus; HO primer pair for HO-induced telomere. Primers used in this study are listed in Supplementary Table S2. Files containing further data (e.g. primer optimization and melting curves) regarding compliance with Minimum Information for Publication of Quantitative Real-Time PCR Experiments (MIQE) are available from the investigators.

### ChIP-dot blot

Relative quantification of telomeric TG repeat sequences in ChIP samples was analyzed by dot-blot (38). ChIP samples and Input DNA were blotted onto positively-charged nylon membrane (GE healthcare Hybond-XL) using a 96-well dot blot manifold and probed with radiolabeled DNA probe against telomeric TG repeats. Signals were assessed by Phosphorimager analysis (Fujifilm FLA-2000), and ChIP signal expressed either as a percentage of signal from Input DNA. ‘Telomere length-corrected’ ChIP values (Supplementary Figure S7) were calculated by dividing the Input DNA signal value by the factor by which TG length is increased in *rif1–pp1bs*, prior to calculation of ChIP signal. The ‘per telomere’ value obtained was normalized to the value in *RIF1-Myc* (39,40).

### Yeast two-hybrid assay

Yeast two-hybrid assay was performed as previously described (20). Mutagenesis of *RIF1* N-terminal fragment was performed essentially as described (20) to create plasmids pKC011 and pKC013.

### Copy number-based replication timing analysis

Yeast strains were synchronized with 3 μM α-factor and released at 25°C, then collected at intervals for DNA content analysis. Extent of replication was analyzed essentially as described (41). Cells fixed in 70% ethanol were processed for flow cytometry (selected samples shown in Supplementary Figure S5A). Mean DNA content of singlet cells at each time point was used to calculate ‘% bulk replication’ value by applying Gompertz function to the time course data (Supplementary Figure S5B). Suitable time point samples were selected for sequencing, with similar extent of replication in all strains and where only extremely late sequences would remain incompletely replicated. Genomic DNA samples were fragmented by sonication so that majority (∼95% or more) of DNA fragments were between 50–500 bp, and the mean length between 200–300 bp. 233 ng of fragmented genomic DNA was used for library construction for Illumina sequencing. Indexed genomic DNA libraries were prepared using NEBNext Ultra II library prep kit for Illumina without size selection, and NEBNext Multiplex Oligos for Illumina using four cycles of amplification (NEB), followed by two rounds of clean-up using Agencourt AMPure XP beads (Beckman Coulter). Library samples were quantified by qPCR using NEBNext Library Quant Kit for Illumina (NEB) and a Rotor-Gene real time PCR cycler (Qiagen). Fragment sizes were confirmed by Tapestation (Agilent). Library samples were mixed in an equimolar ratio and diluted to 2.3 pM for single end deep sequencing by NextSeq 500 using a NextSeq 500/550 High Output v2 kit (75 cycles) (both Illumina) generating an average of 30 million reads per sample.

Relative copy number of the genomic DNA sequence was calculated for 1 kb bins, based on the number of unique mapped sequencing reads (41). Sequencing reads were mapped onto sacCer3 using Bowtie2 (version 2.2.5), reads mapping to a single genomic location were summed in 1 kb windows using Samtools (version 1.3.1) and Bedtools (version 2.26.0). In the R environment, reads from replicating samples were made relative to non-replicating samples processed in parallel, and corrected for differences in read number. The resulting ratios were normalized by the ‘% bulk replication’ value at the corresponding time point obtained by flow cytometry analysis as above (41). Relative copy number was then plotted against sequence location for the appropriate genotypes.

Raw fastq files and processed wig files giving the final calculated relative copy number are available from the NCBI GEO database (accession number GSE109241).

## RESULTS

### A Rif1 mutant that cannot recruit PP1 causes telomere elongation

Because Rif1 acts in replication control as a PP1-targeting subunit, we tested whether ablating Rif1 interaction with PP1 affects telomere length. We previously identified four PP1 interaction motifs located in the N-terminal part of Rif1, which mediate the Rif1–PP1 interaction relevant for replication control (20). We examined telomere length in a strain carrying a *RIF1* allele mutated at these PP1 interaction motifs and encoding a ‘Rif1–pp1bs’ protein that cannot bind to PP1 (*rif1–pp1bs*; Figure 1A) (20). We found that the telomeric terminal TG repeats are elongated by around 600 bp in the *rif1–pp1bs* mutant when compared to wild-type (Figure 1B, lanes 1 and 3–5), resulting in TG repeat sequences of similar length to those in a *rif1Δ* strain (lane 2). This observation is consistent with the hypothesis that PP1 is the mediator of telomere length regulation by Rif1. Addition of a Myc epitope tag to the C-terminus of *RIF1* does not affect its function in telomere length regulation (Figure 1B, lanes 7 and 9–11), confirming suitability of the tagged allele for further experiments. Western analysis showed that the Rif1–pp1bs-Myc protein is expressed at a similar level to a Rif1-Myc (Figure 1C), indicating that the telomere extension in the *rif1–pp1bs* alleles is not due to reduced protein expression.

To confirm the effects of our *rif1–pp1bs* allele and examine the requirement for the four PP1 interaction motifs, we used a two-hybrid assay to test for interaction between PP1 and a Rif1 fragment with two, three or all four of the N-terminal PP1 interaction motifs mutated (Supplementary Figure S1). A Rif1 construct lacking all four PP1 interaction motifs showed no interaction with PP1 (Supplementary Figure S1B upper panel, row 6), while a construct with motifs ‘a’ and ‘b’ ablated but ‘c’ and ‘d’ intact retains some ability to bind PP1 (Supplementary Figure S1B upper panel, row 4). We therefore suspect that it is important to mutate all four N-terminal motifs to fully uncover the roles for Rif1–PP1 interaction.

### PP1 interaction-defective Rif1 binds normally to telomeres

**Figure 2.**
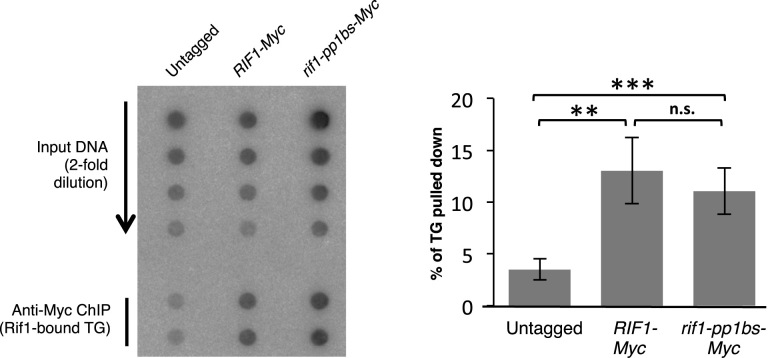
A Rif1–pp1bs-Myc mutant that is unable to interact with PP1 binds to telomeres at comparable levels to wild-type Rif1-Myc. ChIP-dot blot analysis in left panel shows robust recruitment of Rif1–pp1bs-Myc protein to TG sequence. ChIP and Input DNA samples were applied to membrane as a dot blot, and probed for telomeric TG sequence. Right panel shows quantification. The same data adjusted for telomere length and normalized to WT are shown in Supplementary Figure S7. Strains used: SHY201, ASY26 and SMKY30.

We next investigated whether PP1-interaction is required for Rif1 to associate with telomeres. For example, Rif1-associated PP1 might assist Rif1 binding to telomeres by regulating the phosphorylation state of either Rif1 or Rap1. To address whether Rif1–pp1bs does bind normally to telomeres, we compared amounts of TG repeat sequence pulled down by Rif1–pp1bs-Myc or Rif1-Myc in ChIP experiments. Analyzing chromatin immunoprecipitates by probing dot blots with a TG probe, we observed robust pulldown of telomeric TG sequence by both Rif1-Myc and Rif1–pp1bs-Myc (Figure 2, left panel, bottom two rows), implying that the mutated protein is competent for telomere binding. About 12% of the input TG sequence was pulled down by Rif1–pp1bs-Myc, compared with 13% by Rif1-Myc (Figure 2, right panel). This finding demonstrates that PP1 association is not required for telomere association of Rif1. The result also excludes the possibility that lengthened telomeres in the *rif1–pp1bs* mutant are caused by the mutant protein being misfolded and defective for telomere binding.

We used dot blot hybridization with a TG probe to assess Rif1–pp1bs-Myc binding to telomeres (Figure 2A), rather than ChIP-qPCR of a subtelomeric sequence. ChIP-qPCR can produce misleading results when analyzing binding of telomeric factors in strains where telomere lengths differ, probably due to differences in the distance of bound proteins from the amplified probe sequence (which affects the likelihood of any distally located protein being present on the same fragment as the probed sequence after sonication: see Supplementary Figure S2C for illustration). Indeed ChIP-qPCR analysis showed reduced pulldown efficiency of unique sequences close to telomeres VIR or XVL by Rif1–pp1bs-Myc when compared to Rif1-Myc (Supplementary Figure S2A). This difference is likely due to the increased telomere length in the *rif1–pp1bs* strain (and therefore distance of the probe from the Rif1-bound domain), since the pull-down efficiency of unique VIR and XVL sequences was restored in a *rif1–pp1bs tel1Δ* mutant context, in which telomeres are reverted to near-normal length (Supplementary Figure S2A and B).

In summary, we find that ablating Rif1 interaction with PP1 elongates telomeres to virtually the same extent as a complete *RIF1* deletion. The Rif1–pp1bs protein is however fully competent for association with terminal TG repeat sequences, so its effect on telomere length is not caused by compromised telomere association.

### Tethered PP1 can partially substitute for Rif1 in repressing telomere length

**Figure 3.**
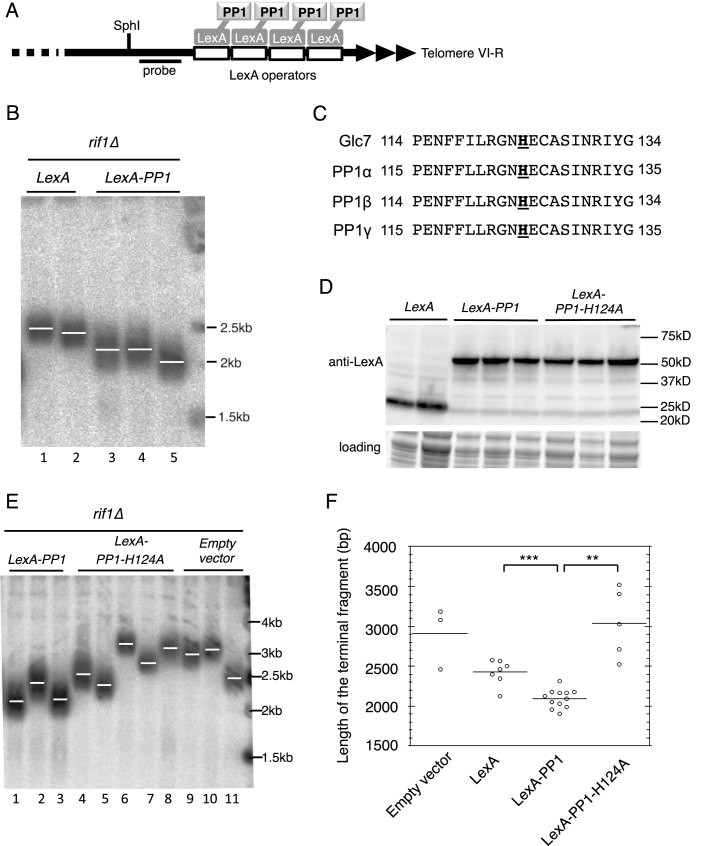
Tethering PP1 at telomere VI-R shortens TG repeat length in the absence of Rif1. (**A**) Schematic illustration of yeast telomere VI-R with 4xLexA operators inserted next to the TG repeats. The terminal *SphI* fragment was detected using the probe indicated. (**B**) In *rif1Δ* background, tethered LexA-PP1 shortens telomere VI-R (lanes 3–5) compared to LexA-only control (lanes 1–2). (**C**) Comparison of catalytic center of PP1 proteins from budding yeast and human. Bold text indicates the histidine residues required for human PP1 activity and the corresponding residue in yeast Glc7. (**D**) Expression of LexA-PP1 fusion proteins confirmed by immunoblotting using LexA antibody. (**E**) PP1 activity is required to shorten telomeres. Catalytically inactive LexA-PP1 protein was tethered to telomere VI-R as in B. Whereas wild-type PP1 shortens telomeres (lanes 1–3), catalytically inactive PP1 does not (lanes 4–8) and the effect is indistinguishable from empty vector control (lanes 9–11). (**F**) Graph showing terminal fragment length of all isolates tested (from parts B and E and not shown), including confirmation that LexA-PP1 causes statistically significant telomere shortening (Student’s *t*-test *** <0.001, ** <0.01). Strains used: SMKY17-21 and SMKY129-133.

The results above suggest that Rif1 may impact telomere length primarily through directing PP1 activity. We therefore tested whether artificial recruitment of PP1 to a specific telomere can substitute for the presence of Rif1 in limiting telomere length. Using a chromosome construct with four LexA binding sites integrated next to telomere VI-right (Figure 3A), we tested the effect of expressing a LexA-PP1 fusion protein, in strains deleted for *RIF1*. We found that, in comparison to a control strain expressing LexA only, the LexA-PP1 protein led to noticeable shortening of the adjacent telomere in all three isolates tested (Figure 3B). The length of other telomeres was not affected (Supplementary Figure S3A). When wild-type Rif1 was present LexA-PP1 did not shorten telomere VI-right (Supplementary Figure S3B), as expected since PP1 recruitment at the TG repeats by Rif1 will be intact and likely to outweigh any effect of the tethered LexA-PP1. This tethering experiment suggested that PP1 recruited next to a telomere does negatively regulate its length, partially compensating for the absence of Rif1.

No catalytically inactive PP1 mutant has previously been described in yeast, so to confirm that phosphatase activity is required for telomere VI-right shortening we considered information from a human PP1 mutant. Mutating residue histidine 125 within the catalytic center of the human PP1-α protein destroys its phosphatase activity (42,43). PP1 is well-conserved especially within this catalytic center region (Figure 3C), so we made the equivalent mutation (H124A) in the yeast LexA-PP1 construct and confirmed its expression in yeast (Figure 3D). We found that this mutated construct was largely incapable of shortening of Tel VI-right (Figure 3E), although effects were somewhat variable between specific isolates. The effects of the various constructs in all replicates tested are plotted in Figure 3F; statistical analysis confirmed significant shortening of telomere VI-right by LexA-PP1 when compared to LexA-PP1-H124A or LexA alone. Overall, the results of these tethering tests support the suggestion that the phosphatase activity of tethered PP1 can partially suppress inappropriate telomere lengthening caused by the absence of Rif1.

While tethering of PP1 in this way does reduce the length of the telomere VI-right terminal fragment, its effect is not as strong as that of endogenous Rif1 (compare Figure 3B, lanes 3–5 with Supplementary Figure S3B). The smaller effect of PP1 tethering may reflect non-optimal positioning of the tethered PP1 relative to the chromosome end, or perhaps that only a limited number of LexA operators were inserted. Attempts to test the effect of a larger number of tethered PP1 fusions were unsuccessful due to the instability of the tethering construct in yeast regardless of fusion protein expression.

**Figure 4.**
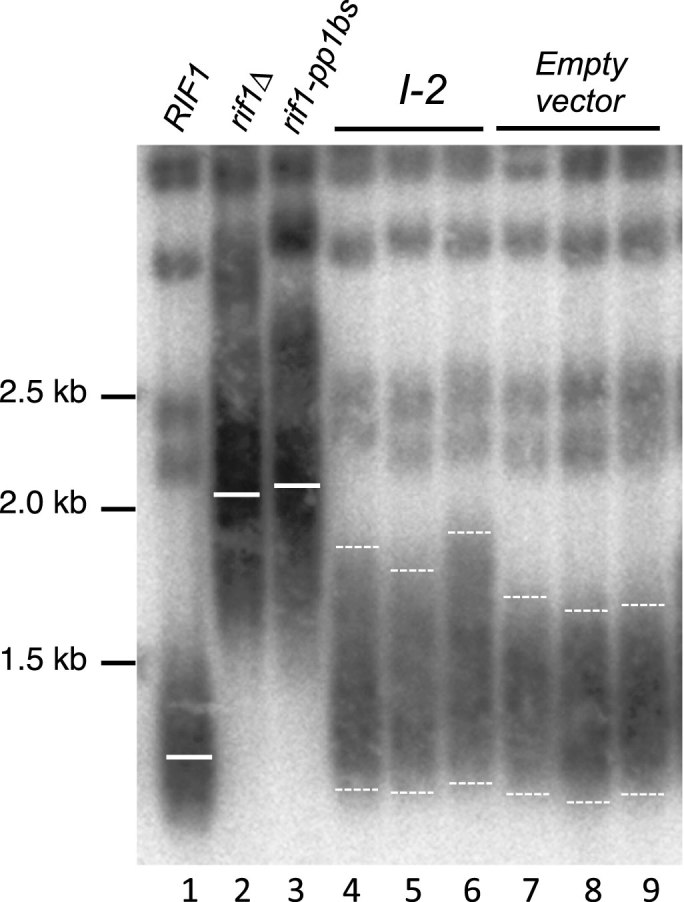
Expression of PP1 inhibitor I-2 in budding yeast causes telomere elongation. Length of telomeres in strains expressing I-2 protein (lanes 4–6) or empty vector (lanes 7–9). Cells were cultivated with I-2 induced for about 100 generations, and telomere length analyzed as in Figure 1B. Strains used: SHY201, SMKY28 and SMKY134–137.

If Rif1 acts with PP1 to prevent telomere over-extension, then we would predict that compromising PP1 activity would cause lengthened telomeres even in the presence of intact Rif1. We therefore examined telomere length in a strain expressing the human PP1 inhibitor protein I-2 (Supplementary Figure S4A), which has been demonstrated also to inhibit activity of the *S. cerevisiae* PP1 protein, Glc7 (44–46). Glc7 is an essential protein but cells were able to grow despite constitutive I-2 expression, suggesting that I-2 inhibits Glc7 phosphatase activity in yeast to an extent where the residual phosphatase activity is sufficient for cell survival. The I-2 expressing cells did nonetheless display mild telomere lengthening with particular elongation of the longest telomeres (Figure 4), consistent with an involvement of PP1 in repressing telomere extension. A mutant in *GLC7* itself showed a similar telomere extension phenotype (Supplementary Figure S4B), again implicating PP1 in telomere length control.

### Rif1 can suppress telomere extension independent of the initiation time of nearby replication origins

Telomere extension normally occurs during S phase concurrent with DNA replication (47), and we wished to investigate whether the effect of Rif1 in telomere length control is connected to its function in controlling replication origin activation time. Rif1–PP1 dephosphorylates the MCM complex to prevent premature activation of its replicative helicase activity, and ablating Rif1–PP1 interaction causes precocious activation of telomere-proximal replication origins and consequent aberrantly early telomere replication. It has several times been suggested that this aberrant early replication favors telomerase recruitment and lengthening. Such a model could potentially explain the regulatory effect of Rif1–PP1 on telomere length, if Rif1 suppresses telomerase recruitment to normal-length telomeres by ensuring their late replication. This model was initially suggested by (8), and discussed in more detailed (19).

**Figure 5.**
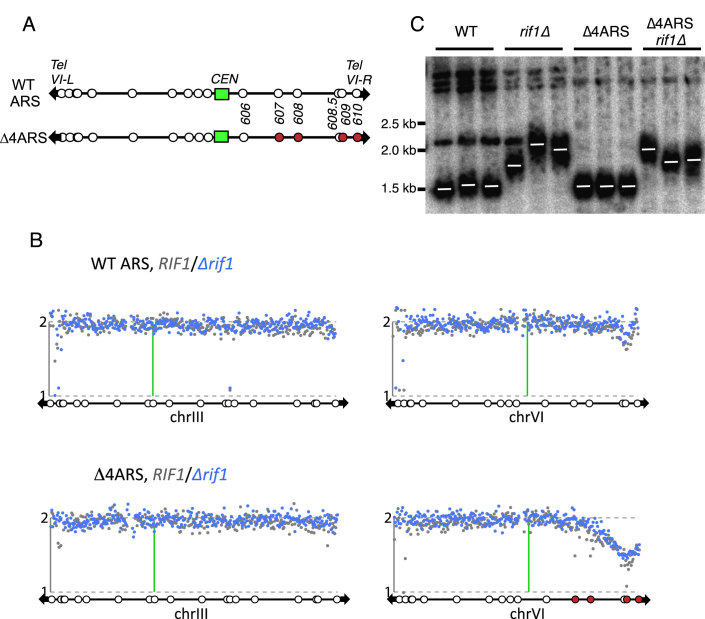
Rif1 does not require nearby origins to affect telomere length. (**A**) Schematic representation of Δ4ARS strains. *ARS* elements on chromosome VI Right (*ARS607, ARS608, ARS609* and *ARS610*; shown in red) are deleted in Δ*4ARS* strains (bottom). (**B**) The Δ4ARS telomere VI-right region replicates very late regardless of *RIF1*. Relative copy number analysis in late S phase of chromosomes III and VI in strains with *ARS607, ARS608, ARS609* and *ARS610* intact (top panels) and Δ4ARS (bottom panels). Gray (*RIF1*) and blue (*rif1*Δ) dots represent the relative copy number of genomic locations (1 kb bins) at 75 min (*RIF1*) or 65 min (*rif1*Δ) after the release from alpha factor arrest at 25°C. See also Supplementary Figure S5A and B. (**C**) *RIF1* can still control telomere VI-R length in Δ4ARS strain, suggesting elongation is not driven by early replication. Genomic DNA samples from strains with intact chromosome VI (WT and *rif1Δ*) were digested with PvuII and probed to detect a specific VI-right terminal fragment whose expected size is 1234 bp plus TG_1–3_ repeats. Genomic DNA samples from Δ4ARS strains (Δ4ARS and Δ4ARS *rif1*Δ) were digested with NcoI and probed with *hphNT1-*specific probe to detect a marker gene within the terminal fragment whose expected size is 1210 bp plus TG_1–3_ repeats. Strains used: GA-1459, SMKY101–103, HE61 and SMKY104–106.

To address whether Rif1 regulates telomere length by ensuring late replication of normal-length telomeres, we used a modified ‘*Δ*4ARS’ strain lacking the four well-characterized replication origins adjacent to chromosome VI-right (*ARS607, ARS608, ARS609* and the X element origin *ARS610*; Figure 5A). It was previously shown that removal of these origins forces abnormally delayed replication of the VI-right terminal region (48). We confirmed that chromosome VI-right is the very last chromosome locus to replicate in both *Δ*4ARS *RIF1* and *Δ*4ARS *rif1*Δ strains (Figure 5B), by genomic copy-number measurement in synchronized cultures at a very late S phase time point (Supplementary Figure S5A and B). If loss of Rif1 causes telomere lengthening through the premature activation of nearby origins, then we would predict that in the *Δ*4ARS context Telomere VI-right would be immune to lengthening upon Rif1 removal, since the absence of nearby origins from Telomere VI-right prevents its early replication. Examining the effect of deleting *RIF1* on the length of telomere VI-right, we found that loss of *RIF1* caused very similar lengthening of the VI-right TG tract in the wild-type and *Δ*4ARS chromosome contexts (Figure 5C). This result implies that Rif1 can suppress telomere lengthening even in a context where it cannot regulate the initiation time of telomere-proximal replication origins.

An additional dormant origin, *ARS608.5*, exists 16.6 kb from Telomere VI-right. Our timing analysis gave no indication that *ARS608.5* becomes active in the absence of *RIF1*, but to make certain that it did not affect the results of these experiments we tested telomere length in a ‘*Δ*5ARS’ strain from which *ARS608.5* was also deleted. Removal of *RIF1* led to similar lengthening of Telomere VI-right in wild-type, *Δ*5ARS and *Δ*4ARS chromosomal contexts (Supplementary Figure S5C), confirming the conclusion that Rif1 can affect telomere length independent of its impact on the initiation time of nearby replication origins.

### Rif1–PP1 suppresses Tel1 recruitment to prevent inappropriate telomere extension

In order to investigate how and when Rif1–PP1 suppresses inappropriate telomere lengthening, we examined the need for Rif1–PP1 interaction in controlling events during telomere elongation. Recruitment of Tel1 kinase is central for stimulating telomerase-mediated TG repeat lengthening ((3) and references therein). Rif1 limits the association of Tel1 with telomeres (5). We therefore investigated whether PP1 mediates the activity of Rif1 in suppressing Tel1 recruitment.

**Figure 6.**
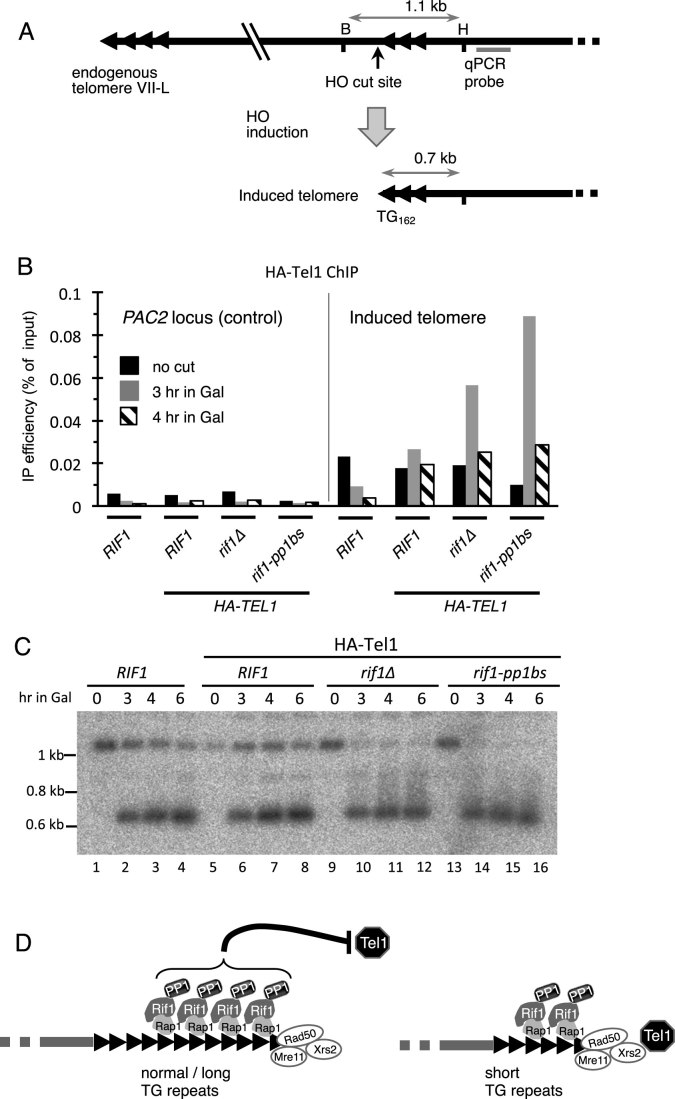
The *rif1–pp1bs* mutation causes Tel 1 kinase recruitment and elongation at a *de novo* telomere induced during a mitotic block. (**A**) Schematic illustration of the HO-induced telomere system. Induction of HO endonuclease by galactose addition cuts next to a 162-bp TG seed sequence to create a *de novo* telomere. Position of the unique sequence detected by real-time qPCR is indicated. (**B**) ChIP-qPCR analysis shows that Rif1–pp1bs protein fails to repress Tel1 recruitment. *HA-TEL1* cells with inducible HO endonuclease were cultivated in sucrose medium, and arrested with nocodazole. HO endonuclease was induced by galactose addition, and anti-HA ChIP samples prepared at indicated time points. Real-time qPCR analysis was performed at a unique DNA sequence near the HO cut site to monitor the recruitment of HA-Tel1 to the newly generated telomere. The *PAC2* locus was analyzed as a control. (**C**) Southern blot analysis reveals abnormal elongation of TG seed sequence in *rif1*Δ and *rif1–pp1bs* mutants. Genomic DNA samples were prepared at indicated time points after the induction of HO endonuclease, cut at HindIII (‘H’) and BsaBI (‘B’) sites, and analyzed by Southern blot probing with a fragment of KanMX that hybridizes immediately to the right of the inserted TG162 sequence. (**D**) At a normal length telomere, sufficient Rif1–PP1 is present to antagonize Tel1 recruitment (left panel). When telomeres are short, insufficient Rif1–PP1 leads to Tel1 recruitment, stimulating elongation by telomerase. Strains used: SMKY91, SMKY93, SMKY95 and SMKY97.

To investigate this possibility we adapted an induced-telomere system (5) in which HO endonuclease cuts next to a 162 bp telomere seed sequence as illustrated (Figure 6A) to produce a *de novo* telomere. The HO cut was induced at cells blocked in mitosis using nocodazole to ensure that events at the *de novo* telomere were uncoupled from DNA replication. Recruitment of HA-tagged Tel1 was monitored by ChIP-qPCR, valid in this experiment because, unlike the situation in Figure 2, the length of the *de novo* telomere is identical in the strains being tested. Three hours after HO endonuclease induction, we found that Tel1 recruitment is increased in *rif1Δ* mutant cells when compared to *RIF1*, as previously described (Figure 6B) (5). We observed a similar enhancement of Tel1 localization to the induced telomere in the *rif1–pp1bs* strain (Figure 6B and Supplementary Figure S6A). This result implies that downregulation of Tel1 recruitment by Rif1 requires PP1 activity, rather then resulting from the mere presence of Rif1 protein.

In this induced-telomere system the absence of Rif1 not only enhances Tel1 recruitment following HO cutting, but also leads to productive telomere extension, visualized as an upward smearing of the terminal 0.7 kb fragment on southern blot analysis (Figure 6C, lanes 10–12). We saw similarly increased extension of the induced telomere in the *rif1–pp1bs* mutant (Figure 6C, lanes 14–16 and Supplementary Figure S6B, last two lanes).

In terms of both Tel1 recruitment and TG repeat extension, the phenotypes of *rif1–pp1bs* therefore resemble those of a *rif1Δ* mutant, consistent with the effect of Rif1 on telomere length control being mediated primarily through PP1. Because these experiments were performed in cells at a mitotic block, the results also confirm that Rif1 can control telomerase-mediated telomere extension independent of effects on telomere replication time during S phase.

## DISCUSSION

In this study we have addressed the long-standing question of how *S. cerevisiae* Rif1 suppresses telomere lengthening, with the discovery that the effect of Rif1 on telomere length depends almost entirely on its interaction with PP1, and that PP1 ectopically recruited to a telomere can partially substitute for Rif1. One recurrent suggestion has been that Rif1–PP1 impacts on telomere length indirectly through its effect on telomere-proximal replication origins. An alternative possibility is that Rif1–PP1 affects telomere length by regulating telomerase recruitment directly. Our results here support this latter possibility, since nearby origins are not needed for Rif1 to affect telomere length (Figure 5). Also, Rif1 can still control elongation even at a telomere created during a mitotic block (Figure 6C), consistent with a previous observation that telomere extension can be controlled separably from S phase (49). The findings we describe here indicate that rather than controlling TG repeat length via replication timing, Rif1–PP1 instead directly suppresses events necessary for telomerase-mediated TG repeat elongation, in particular Tel1 recruitment (Figure 6B and D). Our results support the emerging idea that a central and conserved function of Rif1 is to act as a ‘landing pad’ for PP1 to exert various functions at different chromosomal sites under particular circumstances (19,26,50). It should be noted however that even though ablating Rif1–PP1 interaction almost fully reproduces the effect of deleting Rif1, our results do not exclude the possibility that Rif1 can make a contribution to telomere maintenance and lengthening independent of PP1. Also, although our investigations clearly indicate that Rif1 can control telomere length separately from its regulation of telomere-proximal origin activation (i.e. in the *Δ*4ARS context and in mitotically blocked cells), in normal cells elongation does occur concomitant with telomere replication, and early telomere replication may contribute to telomere lengthening even if not absolutely required.

Our discovery raises the question of what target protein is dephosphorylated by Rif1–PP1 to prevent telomere elongation. Tel1 itself is one good candidate for direct dephosphorylation by Rif1–PP1. Tel1 is a member of the evolutionarily conserved phosphatidylinositol-3-kinase-related family of serine/threonine kinases. Kinase activity that is stimulated by phosphorylation is a general feature of members of this family. For example, Ataxia Telangiectasia Mutated (ATM) kinase (the human homolog of Tel1) is activated by phosphorylation and especially by autophosphorylation at S1981 (51). It therefore seems likely that Tel1 itself is regulated by phosphorylation, and it contains several S/TQ sequences that could potentially be autophosphorylated. However, perhaps because the endogenous levels of Tel1 are extremely low, no study has described control of Tel1 by phosphorylation. We were able to immunoprecipitate endogenous Tel1 (data not shown), but not in sufficient quantity to identify phosphopeptides by mass spectrometry.

While Tel1 presents an interesting potential target for dephosphorylation by Rif1–PP1, various other candidates are possible. Tel1 is recruited to telomeres by interaction with the C-terminus of Xrs2 (10,11), an interaction that could potentially be regulated by Rif1–PP1-mediated dephosphorylation of Xrs2 or even of a different MRX component. The substrate phosphorylated by Tel1 kinase to promote telomere elongation has still not been conclusively identified (2), and could present another logical target for regulation by Rif1–PP1. CDK-mediated phosphorylation is also implicated in controlling telomere elongation (52–54), potentially through phosphorylation of the Cdc13-Stn1-Ten1 complex and a further possibility is that Rif1–PP1 counteracts this CDK-mediated step to limit telomere elongation.

The simplest protein-counting model of Rif1/2 action proposes that more Rif1 binds to long than short telomeres, but there is some uncertainty about whether the amount of Rif1 bound actually changes with telomere length (compare results in (4,11) and (5)). Our ChIP-dot blot analysis (Figure 2) shows a similar percentage of cellular TG sequence pulled down in *rif1–pp1bs* and *RIF1* strains. But given that *rif1–pp1bs* has greatly elongated telomeres, the same percentage of TG sequence pulled down in the two strains in fact tends to suggest there is more Rif1 protein bound at the elongated *rif1–pp1bs* telomeres then the normal-length *RIF1* telomeres (assuming the amount of TG sequence pulled down directly reflects the number of protein molecules bound, as expected). Estimating the relative amounts of Rif1–pp1-Myc and Rif1-Myc protein bound to each telomere (by adjusting the Input TG value of the *rif1–pp1bs* strain for its increased telomere length (39,40)) suggests that around 2-fold more Rif1 protein is bound to telomeres in the *rif1–pp1bs-myc* strain than in *RIF1-myc* (Supplementary Figure S7). Our data therefore are in fact consistent with the possibility that longer telomeres recruit more Rif1 protein as proposed by the protein-counting model. However other interpretations are possible, especially given that we do not know whether during the ChIP procedure telomeric DNA is fragmented like other genomic sequences, or the position and range of Rif1 binding within the TG repeats. An important step forward has been made in understanding how Rif1 acts at telomeres with the recent description of a molecular structure for the conserved HEAT repeats, which form a large N-terminal region of Rif1 (Figure 1A) (55). Unexpectedly, this HEAT domain appears to mediate dimerization of Rif1 and also to directly bind to the telomeric DNA, in a second, Rap1-independent mode of telomere interaction. The authors suggested a model in which Rif1 forms an extended ‘sheath’ that envelops and protects the telomere. Based on the results we present in this study, we propose that as well as protecting the telomere, this extended domain of assembled Rif1 recruits PP1, which then acts to directly control TG repeat lengthening by regulating telomerase-mediated extension.

## DATA AVAILABILITY

Raw fastq files and processed wig files giving the final calculated relative copy number are available from the NCBI GEO database (accession number GSE109241).

## Supplementary Material

Supplementary Data
