# Supplementary material for: Rif1 acts through Protein Phosphatase 1 but independent of replication timing to suppress telomere extension in budding yeast
Source: Nucleic Acids Res. 2018 Feb 26;46(8):3993–4003. doi: 10.1093/nar/gky132 (PMC5934629; doi:10.1093/nar/gky132)
Supplement: Supplementary Data [file gky132_supp.pdf]

# Figure S1

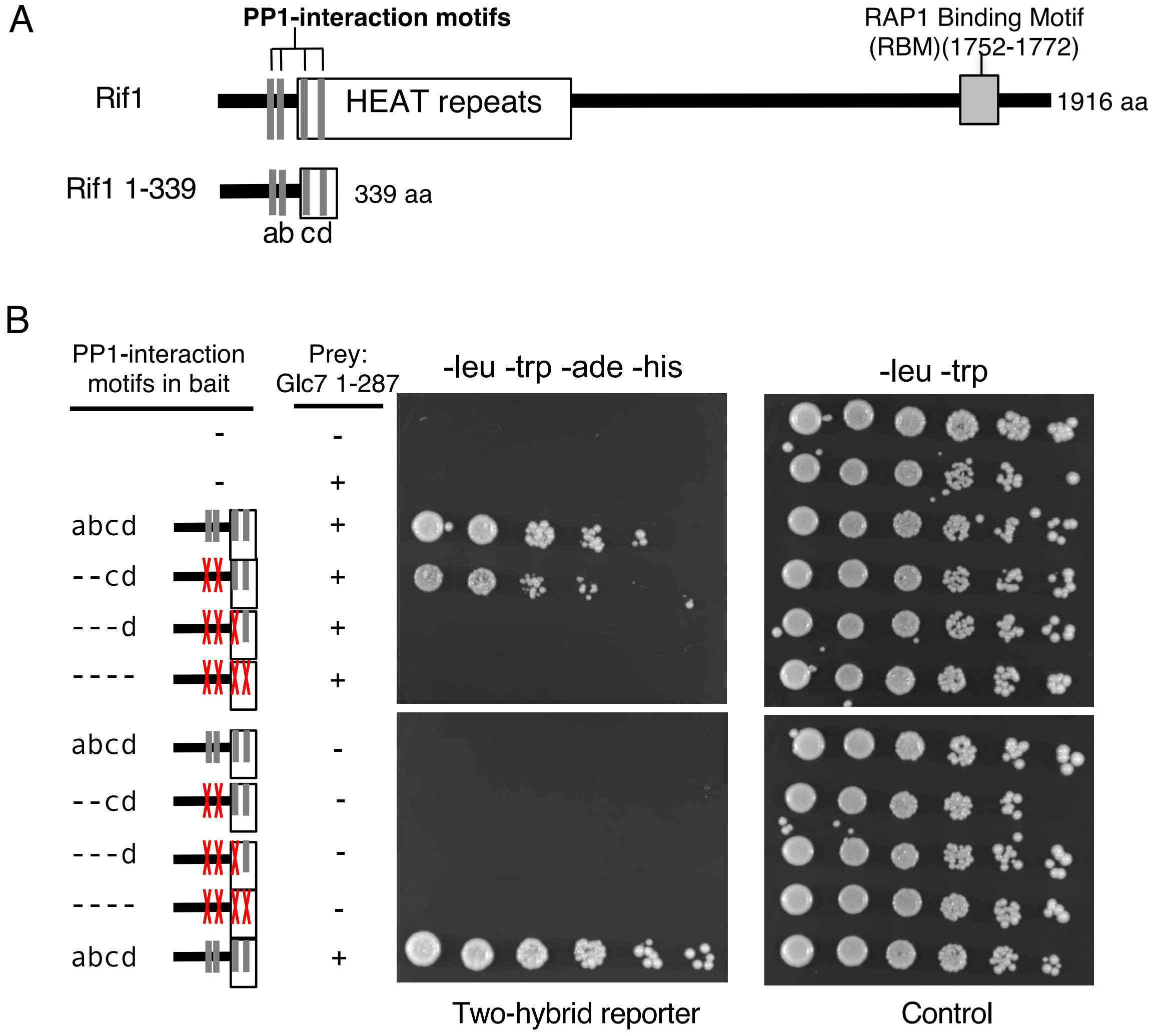
A
PP1-interaction motifs
RAP1 Binding Motif
(RBM)(1752-1772)
Rif1
HEAT repeats
1916 aa
Rif1 1-339
339 aa
ab cd
B
PP1-interaction
motifs in bait
Prey:
Glc7 1-287
-leu -trp -ade -his
-leu -trp
abcd
--cd
---d
----
Two-hybrid reporter
Control

Figure S2

A

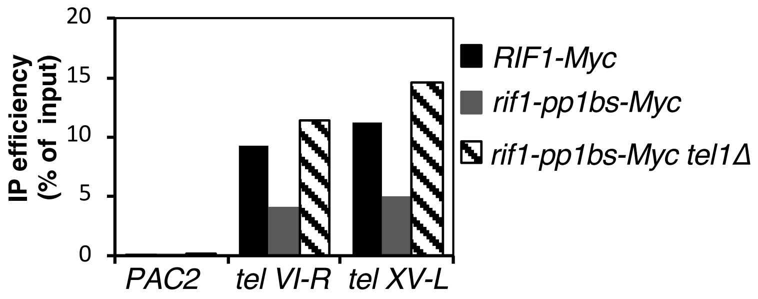


B

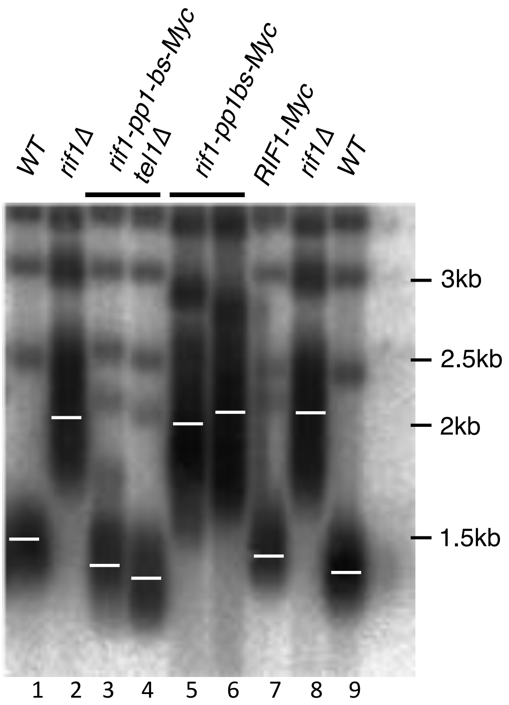


C

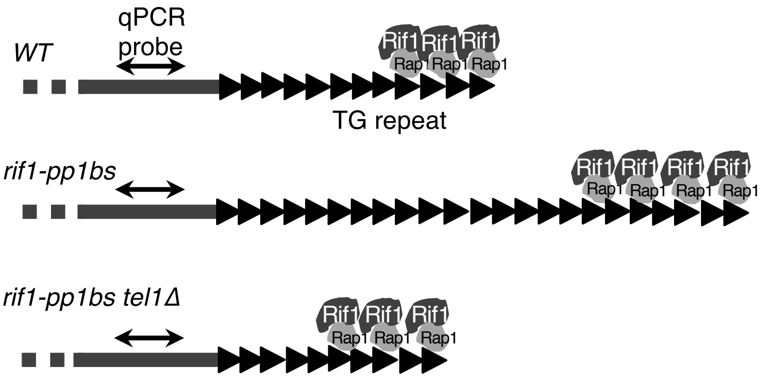

# Figure S3

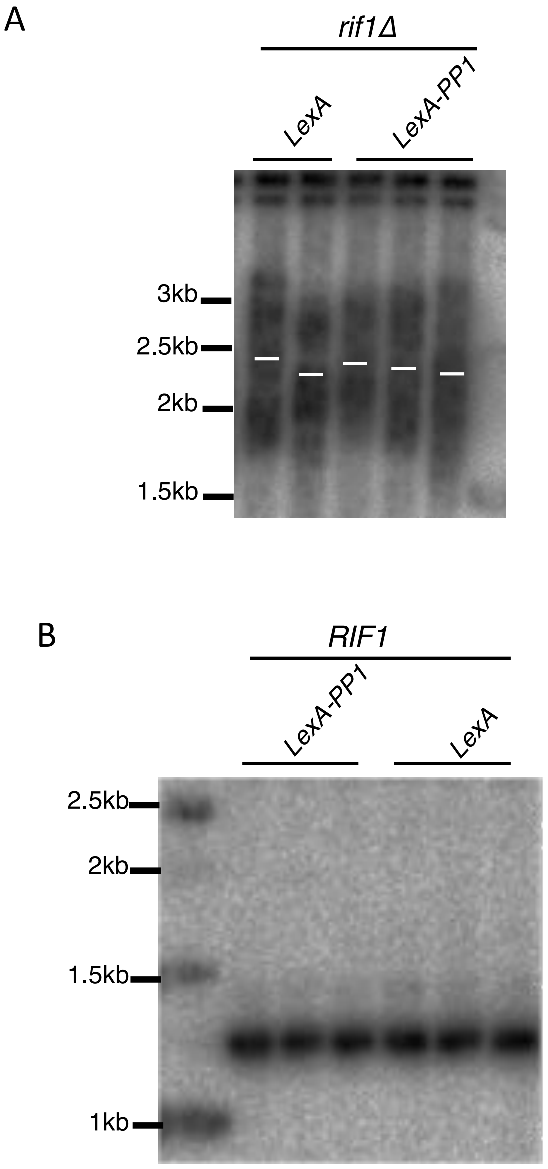
A
rif1Δ
LexA
LexA-PP1
3kb
2.5kb
2kb
1.5kb
B
RIF1
LexA-PP1
LexA
2.5kb
2kb
1.5kb
1kb

Figure S4

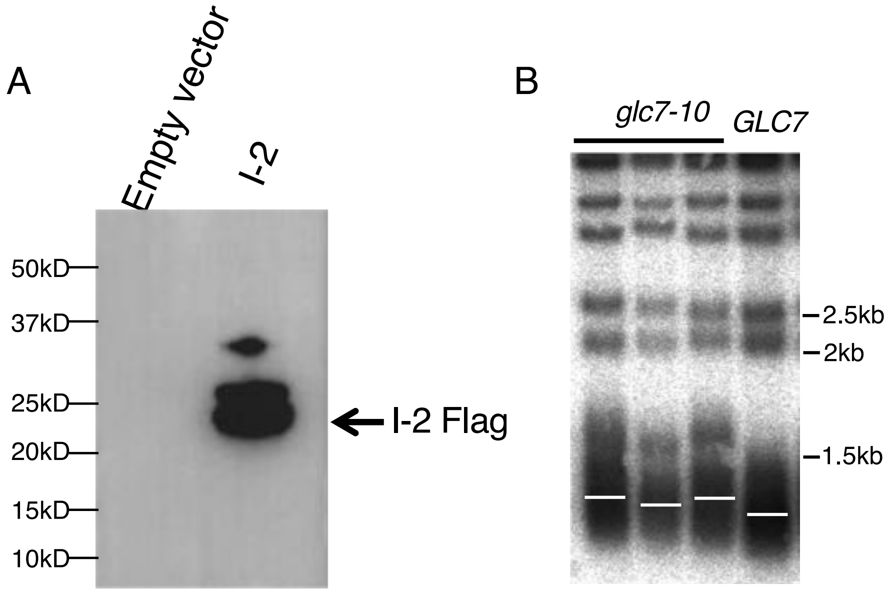
A
Empty vector
I-2
50kD
37kD
25kD
20kD
15kD
10kD
I-2 Flag
B
glc7-10
GLC7
2.5kb
2kb
1.5kb

Figure S5

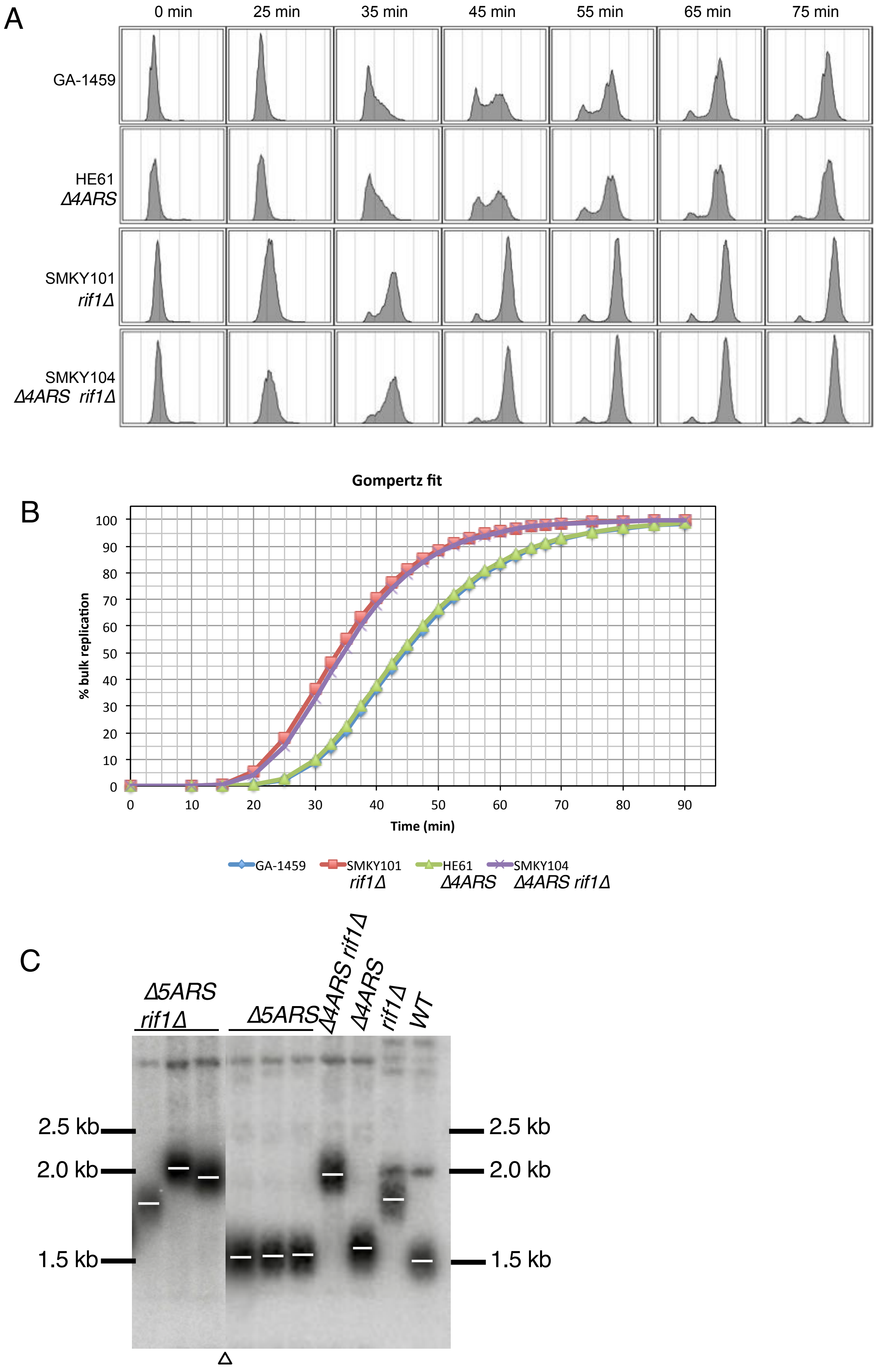

A
0 min
25 min
35 min
45 min
55 min
65 min
75 min
GA-1459
HE61
Δ4ARS
SMKY101
rif1Δ
SMKY104
Δ4ARS rif1Δ
B
Gompertz fit
% bulk replication
Time (min)
GA-1459
SMKY101 rif1Δ
HE61 Δ4ARS
SMKY104 Δ4ARS rif1Δ
C
Δ5ARS rif1Δ
Δ5ARS
Δ4ARS rif1Δ
Δ4ARS
rif1Δ
WT
2.5 kb
2.0 kb
1.5 kb

Figure S6

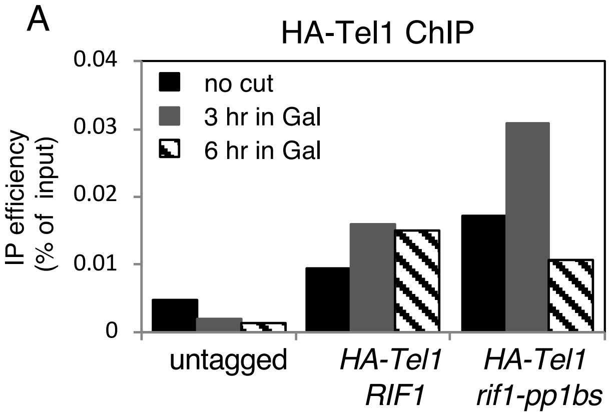
A
HA-Tel1 ChIP
0.04
0.03
0.02
0.01
0
IP efficiency
(% of input)
no cut
3 hr in Gal
6 hr in Gal
untagged
HA-Tel1
RIF1
HA-Tel1
rif1-pp1bs

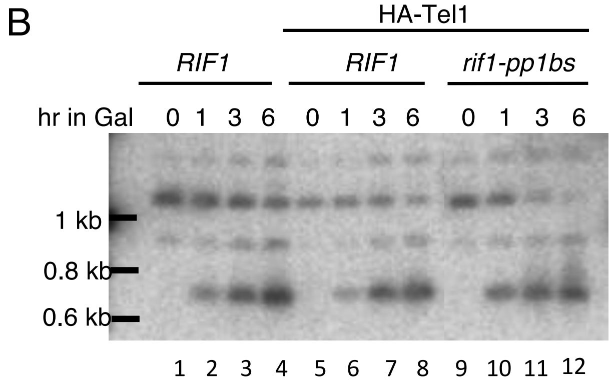
B
HA-Tel1
RIF1
RIF1
rif1-pp1bs
hr in Gal
0 1 3 6 0 1 3 6 0 1 3 6
1 kb
0.8 kb
0.6 kb
1 2 3 4 5 6 7 8 9 10 11 12

Figure S7

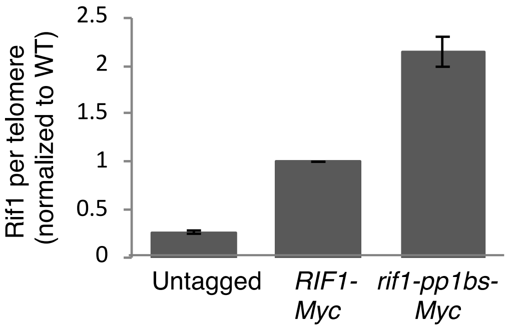

2.5
2
1.5
1
0.5
0
Rif1 per telomere
(normalized to WT)
Untagged
*RIF1-Myc*
*rif1-pp1bs-Myc*

## SUPPLEMENTARY MATERIAL

### Supplementary Figure Legends

**Figure S1. Rif1 interacts with PP1 via four PP1-interaction motifs at its N-terminus**

A. Schematic illustration of Rif1 domains and N-terminal PP1 interaction motifs. An N-terminal Rif1 fragment (amino acids 1-339) containing the four PP1 interaction motifs was used as a bait to analyze the interaction with PP1 (Glc7) by two-hybrid assay.

B. Two-hybrid analysis of interaction between Rif1 N-terminus and Glc7. Double transformant strains with bait and prey plasmids were spotted onto plates lacking adenine and histidine (left) to detect two-hybrid interaction, or plates containing adenine and histidine (right) as a plating control. *ADE2* and *HIS3* are reporter genes in this 2-hybrid system. Bait vector is pGBKT7, while bait plasmids with Rif1 N-terminal fragments are pSH197 (abcd), pKC011 (--cd), pKC013 (---d), and pSH228 (----). Prey plasmids are pGADT7 (empty vector) and pSH197 (Glc7).

**Figure S2. Effect of telomere length on binding of Rif1 as measured by ChIP-qPCR.**

A. Apparent binding efficiency is strongly affected by telomere length when analyzing Rif1 binding by ChIP using qPCR to amplify a subtelomeric DNA fragment. Rif1-Myc and Rif1-pp1bs-Myc ChIP samples were analysed by qPCR with subtelomeric probes against telomeres VI-right and XV-left, in strains with the indicated genotypes. The apparently reduced binding of Rif1-pp1bs-Myc reverts to normal when its elongated telomeres are shortened by deletion of *TEL1*.

B. Telomeres are close to normal in length in *rif1-pp1bs tel1*Δ strain. Telomere length in indicated strains analysed as in Fig 1B.

C. Model illustrating apparent effects of TG repeat length on Rif1 binding as measured using ChIP-qPCR.

Strains used were SHY201, SMKY46, SMKY47, SMKY30, SMKY31, and ASY26.

**Figure S3. Expression of LexA-PP1 does not globally shorten telomeres in a *rif1*Δ background.**

A. Y' telomere length in strains expressing LexA or LexA-PP1. Analyses of two or three independent isolates are shown, in *rif1*Δ strains expressing LexA or LexA-PP1. *Xho*1-digested DNA was probed with a Y'-specific sequence to detect the terminal fragment of Y'-containing telomeres.

B. Length of telomere VI-R is not affected by LexA-PP1 in the presence of intact *RIF1*. *RIF1* strain was transformed with either LexA only or a plasmid expressing LexA-PP1 fusion protein, and the length of telomere VI-R was analysed as in Fig 3B.

Strains used: SMKY17-21, SMKY33-36.

**Figure S4. Compromised PP1 activity lengthens telomeres.**

A. Confirmation of expression human I-2 protein in yeast. A wild-type yeast strain was transformed with either an empty vector or a plasmid with human I-2 gene (PPP1R2) fused to FLAG epitope and under the control of *GAL10* promoter, and protein extracts were prepared after induction by galactose. Expression of I-2-FLAG fusion protein was confirmed by immunoblotting with anti-FLAG antibody.

B. A yeast strain with hypomorphic PP1 allele (*glc7-10*) shows mild telomere elongation. Genomic DNA samples were prepared from *GLC7* and *glc7-10* strains cultivated at 34°C, and length of Y' telomeres was analysed as in Fig 1B.

Strains used: SMKY134, SMKY136, SHY559, and SHY557.

**Figure S5. S phase progression analysis and confirmation that *ARS608.5* does not mediate telomere length regulation**

A. Flow cytometry analysis of S-phase progression in cultures used for replication timing analysis. Cells were blocked with 3μM of alpha factor and released by adding Pronase at 25°C, and cells were collected at 10, 5, or 2.5 min intervals as they progressed through S phase. DNA content profiles of selected time points are shown.

B. Assessment of bulk DNA replication during the time course. Gompertz function was applied to mean DNA content values of the flow cytometry samples, in order to obtain asymmetric sigmoidal functions. '% bulk replication' values for each time point were estimated from the best-fit functions obtained.

C. Deletion of *RIF1* leads to extension of telomere VI-R in the absence of any nearby ARS sequence. Length of telomere VI-R in wild-type, Δ4ARS, and Δ5ARS strains were analysed in *RIF1* and *rif1*Δ backgrounds as in Fig 5C. Open triangle indicates position where irrelevant lanes were removed.

Strains used: GA-1459, HE61, SMKY101, SMKY104, SMKY118-119, SMKY122-123

**Figure S6. Confirmation of effects on HA-Tel1 recruitment and telomere extension at a *de novo* telomere**

HO cut for de novo telomere induction experiment, repeated as in Fig 6.

A. ChIP analysis of HA-Tel1 recruitment to de novo telomere.

B. Analysis by Southern blotting of extension occurring at de novo telomere.

**Figure S7. Relative abundance of Rif1-Myc and Rif1-pp1bs-Myc per telomere.** ChIP-dot blot results presented in Fig 2A, presented as relative amount of Rif1 protein per telomere. The Input DNA value was adjusted for the increased TG repeat length in rif1-pp1bs-Myc, to allow estimation of relative amount of Rif1 protein pulled down per telomere (expressed normalized to value for *RIF1-Myc*). See text for details.

**Supplementary Table S1: Strains used in this study**

| Strain name | Relevant genotype | Figure | Reference |
|---|---|---|---|
| BY4741 | *MATa his3Δ1 leu2Δ0 met15Δ0 ura3Δ0* | n/a | Brachmann et al. 1998 |
| *rif1Δ* | *rif1Δ* (BY4741) | 1B, S2B | Euroscarf |
| SHY201 | *arg4Δ::natMX4 his3Δ1 leu2Δ0 lys2Δ0 ura3Δ0* (BY4741) | 1B, 1C, 2A, 4 S2B | Hiraga et al. 2014 |
| ASY26 | SHY201 *Rif1-13Myc::HIS3* (BY4741) | 1B, 1C, 2A, S2A, S2B | Sridhar et al. 2014 |
| SMKY28 | SHY201 *rif1-pp1bs* Isolate 1 (BY4741) | 1B, 1C, 4 | This study |
| SMKY29 | SHY201 *rif1-pp1bs,* Isolate 2 (BY4741) | 1B | This study |
| SMKY30 | ASY26 *rif1-pp1bs-13Myc::HIS3*, Isolate 1 (BY4741) | 1B, 1C, 2A, S2A, S2B | This study |
| SMKY31 | ASY26 *rif1-pp1bs-13Myc::HIS3*, Isolate 2 (BY4741) | 1B, S2B | This study |
| SMKY46 | SMKY30 *rif1-pp1bs-13Myc::HIS3, tel1Δ:KanMX,* Isolate 1 (BY4741) | S2A, S2B | This study |
| SMKY47 | SMKY30 *rif1-pp1bs-13Myc::HIS3, tel1Δ:KanMX,* Isolate 2 (BY4741) | S2B | This study |
| SMKY16 | *rif1Δ*<br>4xLexA operators next to telomere VI-R | n/a | This study |
| SMKY17 | SMKY16 [pSMK2 ], Isolate 1 (BY4741) | 3B, S3 | This study |
| SMKY18 | SMKY16 [pSMK2 ], Isolate 2 (BY4741) | 3B, S3 | This study |
| SMKY19 | SMKY16 [pSMK2 ], Isolate 3 (BY4741) | 3B, S3 | This study |
| SMKY20 | SMKY16, [pAT4], Isolate 1 (BY4741) | 3B, S3 | This study |
| SMKY21 | SMKY16, [pAT4], Isolate 2 (BY4741) | 3B, S3 | This study |

| | | | |
|---|---|---|---|
| SMKY32 | *BY4741*<br>4xLexA operators next to telomere VI-R | n/a | This study |
| SMKY33 | SMKY32 [pAT4], Isolate 1 | S3B | This study |
| SMKY34 | SMKY32 [pAT4], Isolate 2 | S3B | This study |
| SMKY35 | SMKY32 [pSMK2], Isolate 1 | S3B | This study |
| SMKY36 | SMKY32 [pSMK2], Isolate 2 | S3B | This study |
| SMKY128 | SMKY16, [pRS425], Isolate 1 (BY4741) | 3E | This study |
| SMKY129 | SMKY16, [pRS425], Isolate 2 (BY4741) | 3E | This study |
| SMKY130 | SMKY16, [pRS425], Isolate 3 (BY4741) | 3E | This study |
| SMKY131 | SMKY16 [pSMK5], Isolate 1 (BY4741) | 3D-F | This study |
| SMKY132 | SMKY16 [pSMK5], Isolate 2 (BY4741) | 3D-F | This study |
| SMKY133 | SMKY16 [pSMK5], Isolate 3 (BY4741) | 3D-F | This study |
| SHY559 | *MATa ade2-1 leu2-3,112 his3-11,15*<br>*trp1-1::glc7-10::TRP1 ura3-1*<br>*glc7Δ::LEU2*<br>*MCM4-6His-3FLAG::nat1*<br>*can1-100 ssd1-d2 Gal*$^{+}$ (W303) | S4B | Hiraga et al. 2014 |
| SHY557 | *MATa ade2-1 leu2-3,112 his3-11,15*<br>*trp1-1::GLC7::TRP1 ura3-1 glc7Δ::LEU2*<br>*MCM4-6His-3FLAG::nat1*<br>*can1-100 ssd1-d2 Gal*$^{+}$ (W303) | S4B | Hiraga et al. 2014 |
| GA-1459 | *NUP49-GFP*<br>*his3-11,15::HIS3p-GFP-lacI-HIS3*<br>*ARS609::ARS609 (lacO)-TRP1*<br>*ura3 leu2 trp1-1* (W303) | 5B, 5C, S5A-C | Hediger et al. 2002 |
| SMKY101 | GA-1459<br>*rif1Δ::NAT,* Isolate 1 (W303) | 5B, 5C, S5A-C | This study |
| SMKY102 | GA-1459<br>*rif1Δ::NAT,* Isolate 2 (W303) | 5C | This study |
| SMKY103 | GA-1459<br>*rif1Δ::NAT*, Isolate 3 (W303) | 5C | This study |

| | | | |
|---|---|---|---|
| HE61 | GA-1459<br>*ARS609::ARS609 (lacO)-TRP1*<br>*ARS607::URA3, ARS609::LEU2,*<br>*ARS610::hphNT1, ARS608::kanMX* (W303) | 5B, 5C, S5A-C | Ebrahimi et al. 2008 |
| SMKY104 | HE61<br>*rif1Δ::NAT,* Isolate 1 (W303) | 5B, 5C, S5A-C | This study |
| SMKY105 | HE61<br>*rif1Δ::NAT,* Isolate 2 (W303) | 5C | This study |
| SMKY106 | HE61<br>*rif1Δ::NAT,* Isolate 3 (W303) | 5C | This study |
| SMKY134 | SHY201 [pYES2], Isolate 1 (S288C) | 4, S4A | This study |
| SMKY135 | SHY201 [pYES2], Isolate2 (S288C) | 4 | This study |
| SMKY136 | SHY201[pESC-URA-PPP1R2], Isolate 1 (S288C) | 4, S4A | This study |
| SMKY137 | SHY201[pESC-URA-PPP1R2], Isolate 2 (S288C) | 4 | This study |
| H821 | *VII-L::kanMX-TG162-HO* | n/a | Hirano et al. 2009 |
| H855 | *VII-L::kanMX-TG162-HO MRE11-myc::TRP1 TEL1-HA::TRP1* | n/a | Hirano et al. 2009 |
| H1013 | *VII-L::kanMX-TG162-HO rif1Δ::URA3 MRE11-myc::TRP1 TEL1-HA::TRP1* | n/a | Hirano et al. 2009 |
| SMKY91 | H821 [YCpA-Gal-HO] | 6B, 6C, | This study |
| SMKY93 | H855 [YCpA-Gal-HO] | 6B, 6C, | This study |
| SMKY95 | H1013 [YCpA-Gal-HO] | 6B, 6C | This study |
| SMKY97 | *VII-L::kanMX-TG162-HO rif1-pp1bs*<br>*MRE11-myc::TRP1 TEL1-HA::TRP1*<br>[YCpA-Gal-HO] | 6B, 6C, | This study |
| SMKY118 | *HE 61,*<br>*ARS608.5::ADE2,* Isolate 1 | S5C | This study |
| SMKY119 | *HE 61,*<br>*ARS608.5::ADE2,* Isolate 2 | S5C | This study |
| SMKY122 | *SMKY104,*<br>*ARS608.5::ADE2,* Isolate 1 | S5C | This study |
| SMKY123 | *SMKY104,*<br>*ARS608.5::ADE2*, Isolate 3 | S5C | This study |

## Supplementary Table S2: Plasmids used in this study

| Plasmid name | Description | Reference |
|---|---|---|
| pAT4 | Empty vector for LexA fusion.<br>LexA is under the control of *ADH* promoter and terminator. | Taddei et al. 2004 |
| pSMK2 | pAT4-LexA-GLC7 fusion for GLC7 tethering | This study |
| pSMK5 | pAT4-LexA-GLC7 H124A fusion for GLC7 H124A tethering | This study |
| YCpA-Gal-HO | HO endonuclease under Gal promoter | Nakada et al. 2003 |
| pHL016 | Contains wild type *RIF1* | Hiraga et al. 2014 |
| pSH192 | Contains *rif-pp1bs* mutant | Hiraga et al. 2014 |
| pGBKT7 | Bait vector for two hybrid assay | Clontech |
| pSH197 | pGBKT7 with *RIF1* N-terminal fragment (amino acids 1-339); (abcd) | Hiraga et al. 2014 |
| pKC011 | pSH197 with PP1-binding motifs a and b mutated (--cd) | This study |
| pKC013 | pSH197 with PP1-binding motifs a, b, and c mutated (---d) | This study |
| pSH228 | pSH197 with PP1-binding motifs a, b, c, and d mutated (----) | This study |
| pGADT7 | Prey vector for two-hybrid assay | Clontech |
| pSH220 | pGADT7 with full length *GLC7* cDNA (1-287 amino acids) cloned | Hiraga et al. 2014 |
| pRS425 | Empty vector control for LexA-PP1 tethering system | Christianson et al., 1992 |
| pYES2 | Empty vector control for I-2 | |
| pESC-URA-PPP1R2 | Yeast vector expressing human I-2 protein under Gal promoter | This study |

## Supplementary Table S3: List of primers

| Primer name | Sequence 5'-3' | Target | Reference |
|---|---|---|---|
| M1367 (=SMK78) | AATAACGAATTGAGCTATGACACCAA | *PAC2* | Holstein et al. 2014 |
| M1368 (=SMK79) | AGCTTACTCATATCGATTTCATACGACTT | *PAC2* | Holstein et al. 2014 |
| M2245 (=SMK80) | CGTATGCTAAAGTATATATTACTTCACTCCATT | *chrVI-R* | Holstein et al. 2014 |
| M2246 (=SMK81) | TCCGAACTCAGTTACTATTGATGGAA | *chrVI-R* | Holstein et al. 2014 |
| 15L-fwd (=SMK86) | GGGTAACGAGTGGGGAGGTAA | *chrXV-L* | Balk et al. 2013 |
| 15L-rev (=SMK87) | CAACACTACCCTAATCTAACCCTGT | *chrXV-L* | Balk et al.2013 |
| SMK53 | CTGGCTGGAATTCCCGATGGACTCACAACCAGTTGAC | *LexA-GLC7* | This study |
| SMK54 | AGGTCGACGGATCCCCTTATTTTTCTTTCTACCCCCA GCTTG | *LexA-GLC7* | This study |
| SMK71 | GGAAGTTATAAGTTCATCAATAAAATTC | *S.pombe his3* | This study |
| SMK72 | ATGGGTAGGAGGGCTTTTG | *S.pombe his3* | This study |
| HO (=SMK118) | GTTGTTTCTGAAACATGGCAAAGG | *kanMX* | Hirano et al.2009 |
| HO (=SMK119) | CAACCAAACCGTTATTCATTCGTG | *kanMX* | Hirano et al. 2009 |
| SMK123 | CTCACCGGATTCAGTCGTC | *KanMX* | This study |
| SMK125 | CTCTCACATCTACCTCTACTCTC | *KanMX* | This study |
| SMK154 | GGAAGCACATTCAgcGTTCCCTCTTAAAATGAAAAA | *LexA-GLC7 (PP1) H124A* | This study |
| SMK155 | TTAAGAGGGAACgcTGAATGTGCTTCCATTAATAG | *LexA- GLC7 (PP1) H124A* | This study |

**References:**


Balk, B., Maicher, A., Dees, M., Klermund, J., Luke-Glaser, S., Bender, K., et al. (2013). Telomeric RNA-DNA hybrids affect telomere-length dynamics and senescence. *Nature Structural & Molecular Biology,* **20**, 1199-1205.

Brachmann C.B., Davies, A., Cost, G.J., Caputo, E., Li, J., Hieter, P., & Boeke, J.D. (1998). Designer deletion strains derived from Saccharomyces cerevisiae S288C: a useful set of strains and plasmids for PCR-mediated gene disruption and other applications. *Yeast*, **14**, 115-132

Christianson, T. W., Sikorski, R. S., Dante, M., Shero, J. H., & Hieter, P. (1992). Multifunctional yeast high-copy-number shuttle vectors. Gene, **110** 19-122.

Ebrahimi, H. and Donaldson, A.D. (2008) Release of yeast telomeres from the nuclear periphery is triggered by replication and maintained by suppression of Ku-mediated anchoring. *Genes Dev*, **22**, 3363-3374.

Hediger, F., Neumann, F.R., Van Houwe, G., Dubrana, K. and Gasser, S.M. (2002) Live imaging of telomeres: yKu and Sir proteins define redundant telomere-anchoring pathways in yeast. *Curr Biol*, **12**, 2076-2089.

Hiraga, S., Alvino, G.M., Chang, F., Lian, H.Y., Sridhar, A., Kubota, T., Brewer, B.J., Weinreich, M., Raghuraman, M.K. and Donaldson, A.D. (2014) Rif1 controls DNA replication by directing Protein Phosphatase 1 to reverse Cdc7-mediated phosphorylation of the MCM complex. *Genes Dev*, **28**, 372-383.

Hirano, Y., Fukunaga, K. and Sugimoto, K. (2009) Rif1 and Rif2 inhibit localization of Tel1 to DNA ends. *Mol Cell*, **33**, 312-322.

Holstein, E. M., Clark, K. R., & Lydall, D. (2014). Interplay between nonsense-mediated mRNA decay and DNA damage response pathways reveals that Stn1 and Ten1 are the key CST telomere-cap components. *Cell Reports,* **7**, 1259-1269.

Nakada, D., Matsumoto, K., & Sugimoto, K. (2003). ATM-related Tel1 associates with double-strand breaks through an Xrs2-dependent mechanism. *Genes & Development,* **17**, 1957-1962.

Sridhar, A., Kedziora, S. and Donaldson, A.D. (2014) At short telomeres Tel1 directs early replication and phosphorylates Rif1. *PLoS genetics*, **10**, e1004691.

Taddei, A., Hediger, F., Neumann, F.R., Bauer, C. and Gasser, S.M. (2004) Separation of silencing from perinuclear anchoring functions in yeast Ku80, Sir4 and Esc1 proteins. *EMBO Journal*, **23**, 1301-1312.
